# Depathologising the university

**DOI:** 10.1080/14681366.2024.2316007

**Published:** 2024-02-12

**Authors:** Dan Goodley

**Affiliations:** iHuman, University of Sheffield, Sheffield, UK

**Keywords:** depathologisation, university, equality, diversity and inclusion, decolonisation

## Abstract

This paper develops a conversation with decolonisation to pitch a novel mode of engagement; depathologising the university. While higher education institutions are in the midst of an Equality, Diversity and Inclusion revolution, I posit that all is not well. Too often disability staff and students have been sidelined in Equality, Diversity and Inclusion discourse and practice and this paper addresses this omission. First, I pose a question ‘what is the university for?’ and consider two recent campaigns by Black and Minority Ethnic and disabled students in the UK that offer partial responses to this question. I argue that these campaigns not only implicate the colonial and ableist heritage of universities but also illuminate two critical modes of engagement: decolonisation and depathologisation. Second, to focus the discussion, I introduce *Disability Matters*; a new six year programme of research which seeks to promote more inclusive university environments through positioning disability as the driving subject of inquiry. Third, I offer some provisional and anticipatory thoughts by sitting with decolonisation in order to expand upon a project of depathologisation. I conclude with an appeal; desiring disability’s disruptive qualities to rethink the university.

## Introduction

Equality, Diversity and Inclusion (EDI) has become a core business for universities, and we are witnessing more and more interventions to promote positive university cultures, policies and practices. Too often, however, disabled academics, professional service colleagues and students are sidelined (Brown and Leigh 2020). This paper seeks to address this omission. I begin with a premise; all is not well in the current clamour for EDI. As Lett et al (2022, 1) write:

Diversity. Equity. Inclusion. Anti-Racism. Intersectionality. These are words with rich meanings, theoretical traditions, and scholarly legacies that are meant to inform the practice of pursuing cross-disciplinary justice, grassroots organizing, political advocacy, and scientific inquiry. Recently, they have also become buzzwords that have been shuffled into seemingly meaningless acronyms at healthcare institutions and research organizations.

Lett et al. (2022, 1) argue that research contexts risk being overtaken by ‘health equity tourists’ – bureaucrats who pollute equity landscapes with ineffectual harmful studies and dilute more radical practices as they outnumber community members who have historically built critical epistemologies, ontologies and methodologies. Lett et al’.s words of caution apply to universities; not least their argument that real cultural transformation can only ever be enacted when diverse and activist community members are leading these changes. Unless those people who are intimately connected with human diversity are at the centre of EDI knowledge production then we risk recreating forms of equity tourism in our universities.

Universities feel out of reach to certain members of our local, national and international communities. This reality is at odds with the aspirations of the *civic university* that serves the needs of its local, national and international communities (Wood 2019). Many institutions sell themselves as civic universities while also competing with other institutions for students, research income and global rankings. Universities are also governed, surveilled, assessed and evaluated by their national governments and accountable to the public, their students, their staff and the wider higher education market (Lalu 2019). British universities, for example, juggle competing forms of accountability as they are evaluated in terms of their research (REF: Research Excellence Framework), education (TEF: Teaching Excellence Framework) and impact (KEF: Knowledge Excellence Frameworks). Moreover, universities are compromised by their histories. Following Lalu (2019), we can ask *what* the university does and *who* the university is for. If you are a person of colour, a working class, queer or disabled person you might well ask yourself, is the university for me? Many universities, especially those in Western Europe and North America (WENA), are grappling with their entangled histories of colonialism, racism, ableism and disablism.

This paper adopts a discursive and exploratory approach to consider the challenges of EDI in the university. First, I pose a question ‘what is the university for?’ and consider two recent campaigns by Black and Minority Ethnic and disabled students that offer partial responses to this question. I argue that these campaigns not only implicate the colonial and ableist heritage of universities but also illuminate two critical modes of engagement: decolonisation and depathologisation. Second, to focus the discussion, I introduce *Disability Matters*; a new six year programme of research which seeks to promote more inclusive university environments through positioning disability as the driving subject of inquiry. Third, I offer some provisional and anticipatory thoughts in relation to the study of the inclusive university, by sitting with decolonisation in order to expand upon a project of depathologisation. This latter mode of engagement, I suggest, offers a unique and significant framework that appeals to disability’s disruptive qualities to rethink the university; an argument I pick up in the conclusion.

## Coloniality, pathology and the university

The university can often be a very challenging place. I have often experienced imposter syndrome in the academy – as a first generation working class graduate – and yet as a non-disabled, white, cisgendered straight male, I know that the university has been far easier for me to negotiate than it has for some of my colleagues. The British university is a contrary institution.^1^ On the one hand, the university has ‘a practical benefit – will have been a public good’ (Lalu 2019, 46) and on the other it is implicated in colonial conquest,

The nineteenth‐ century university that upheld the Euro‐US episteme may have inadvertently formed its discourse not only on the basis of the ideals of liberalism but on the very racial scripts of culture that sustained the justi‐ficatory structure of colonialism. (Lalu 2019, 46)

We have witnessed a number of recent high profile campaigns, demonstrations and commentaries that have unearthed and challenged the imperial histories of British universities. Let us consider one example,

Oxford students step up campaign to remove Cecil Rhodes statue Campaigners say removing statue of British colonialist is a small part of tackling racism at Oxford and would help address UK’s ‘imperial blind spot’. Oxford students fighting to have a statue of Cecil Rhodes removed from Oriel College have said the row over his legacy demonstrates Britain’s ‘imperial blind spot’ and criticised the university’s record on black and ethnic minorities. The Rhodes Must Fall group last week succeeded in persuading the college to move a plaque dedicated to him and consult on whether to take down his statue from the Grade II-listed building but the students behind it have said they hope to widen their campaign.

Brian Kwoba, a 33-year-old doctoral student at Oxford and one of the campaign’s organisers, said he and fellow students were inspired by recent events in South Africa, when students at the University of Cape Town hurled buckets of excrement and paint over a statue of Rhodes that was eventually removed.

‘Cecil Rhodes is responsible for all manner of stealing land, massacring tens of thousands of Black Africans, imposing a regime of unspeakable labour exploitation in the diamond mines and devising proto-apartheid policies’, Kwoba said. ‘The significance of taking down the statue is simple, Cecil Rhodes is the Hitler of southern Africa. Would anyone countenance a statue of Hitler? The fact that Rhodes is still memorialised with statues, plaques and buildings demonstrates the size and strength of Britain’s imperial blind spot’. (Khomami 2015, np)

The educational theorist Ball (2022) argues that while US debates about systemic racism have been framed by matters of slavery and civil rights, flashpoints in Britain occur around questions of ‘imperialism and colonialism, which involved white Europeans exploiting other ethnic groups, especially through the slave trade and oppression of Indigenous people’ (Ball 2022, 493). Unearthing a university’s colonial past raises questions about identity, belonging and community. Black and minority ethnic (BAME)^2^ academics, students and professional service colleagues often feel alienated in and by their own institutions. As one Black undergraduate student put it, ‘we know this place was never built with us intended to be here … If you’re not here, you’re not meant to be here’ (Ball 2022, 595). University communities can make us feel like we *belong*; *being* in a community and *longing* to be in it (Yuval-Davis 2006). When that community is unwelcoming then we can neither *be* nor *long* to be in the university. In failing to interrogate their colonial pasts, universities risk maintaining white privilege (Housee 2022). McIntosh (2007, 1–3) writes, ‘as a white person, I realized I had been taught about racism as something that puts others at a disadvantage but had been taught not to see one of its corollary aspects, white privilege, which puts me at an advantage’. While critically reflexive accounts such as these are important, narratives of white guilt should not overpower the accounts of BAME colleagues (Margolin and Martiniello, 2015). As a white scholar myself, I know that my own cultural praxis has to be accountable to my BAME colleagues and students. A 2022 special issue of *Nature* entitled ‘Racism, overcoming science’s toxic legacy’ provides a contemporary cultural commentary on white privilege and anti-black racism within the university. The editorial reads,

For centuries, science has built a legacy of excluding people of colour and those from other historically marginalized groups from the scientific enterprise. Institutions and scientists have used research to underpin discriminatory thinking, and have prioritized research outputs that ignore and further disadvantage marginalized people. (Nature 2022a)

The editorial’s mention of institutions has in mind those places where science is practised and knowledge is generated. The university is one such space. In one article of this special issue, Black researchers make the case that fighting racism demands more than words (Nature 2022b). Racism and white privilege remain stubborn problems. Recent twitter hashtags such #BlackInTheIvory have encouraged Black and BIPOC researchers to share their own academic trajectories in spite of systemic racism and unconscious bias. *Nature’s* special issue is populated with accounts of everyday racism that leave Black colleagues demanding radical change (Nature 2022b) – to *decolonise* the university – to create new ecosystems in which BAME academics, scientists, professional service colleagues, students and members of the wider community feel welcome and welcomed. Decolonising the university emphasises systemic change rather than assimilation (Bhambra, Nişancıoğlu, and Gebrial 2020, Lalu 2019, Housee 2022, Zondi 2022, Masitera 2020, Zondi 2022). Systemic change calls for the transformation of universities. Assimilation simply invites people into existing racist environments. Rizvi (2023) argues that racism is threaded into the very DNA of the contemporary university and Gillborn (2006) writes that to simply assert an anti-racist intention means nothing if we leave unchanged the dominant system that underpins the university, institutional racism. Following Gillborn (2006, 21), this means understanding racism as wide-ranging, often hidden and commonplace, generated in the normal and normalised workings of the university. Decolonisation is not an easy or reducible practice.

As Tuck and Yang (2012, 2) argue, ‘when we write about decolonization, we are not offering it as a metaphor; it is not an approximation of other experiences of oppression’. Similarly, Lett et al (2022) worry about the watering down of decolonising practices if and when these practices become yet another agenda item on the ‘task and finish’ groups of universities. White privilege is loaded, endemic and supportive of the idea that if the university works well for white people then it must be working well for all. Puncturing this logic is at the heart of decolonising the university. While BAME colleagues should be the ones leading decolonisation, it is incumbent on white colleagues to support this work. Bhambra et al (2020) describe decolonising the university as a ‘strategic mode of engagement’ through which the university might be reclaimed and/or rebuilt. These scholars worry that decolonisation is too readily being taken up by equity tourists who refashion the university brand but fail to seriously transform the institution at the heart of the marketing campaign. Instead, Bhambra et al (2020, 511) write, decolonising is ‘an attempt to either recover or create a new an alternative vision of the university, one free of colonial legacies, institutional racism and market forces; a university for the public good(s) of critical thinking, educated deliberation and informed citizenship’.

Just as critical responses to the colonial heritage of British universities have been driven by BAME activists, researchers and campaigners, disabled people have also contested the exclusion faced by disabled staff and students,

Disabled students at the University of Birmingham claimed they were being ‘discriminated against’ and ‘failed’ in their education. Student group DAMSA – the Disability and Mental Health Students Association – held a protest on campus demanding equal access to education for people with physical and learning disabilities … At the protest, held on campus on Wednesday, September 28, [2022] around 30 students voiced their anger, shared experiences and demanded action from the university. In the open letter, written by DAMSA, the group said, ‘The University of Birmingham actively harms its disabled students – we are being failed and action needs to be taken’. Among the protestors was Modern Languages student Yas. They said they were denied the opportunity to go on a year abroad this year after the university was unable to arrange suitable accommodation. As a result, Yas – who became a wheelchair user after contracting covid – said they were forced to take a leave of absence against their will …

Clara Gott is a Classics student at the university. She spoke of the difficulties she faced when trying to get around campus. ‘So much of our campus is inaccessible’, she said. ‘There are so many potholes, ridiculous hills and no public transport from Selly Oak … There are buildings you can’t access unless you know the back routes, lifts that don’t work. I’ve been here since 2017 and it’s become worse – I don’t trust the university to do anything’. Yas said the lack of accessibility meant they had no choice but to risk their health to get onto campus. ‘I had no other option than to make myself sick by forcing myself to walk up these hills when I was in Selly Oak’, they said. ‘My grades suffered – I don’t know how I passed last year’. Clara called for more active support for disabled students from the community. She claimed complaints were ‘routinely ignored’ and she felt unwelcome at the university. (Clarke 2023, np)

The university has become a key site for disability politics and scholarship. Brown and Leigh’s (2018, 2020) seminal work demonstrates how the campus, lecture room, curriculum document, reading list, pedagogy and assessment system all presume a particular kind of student or staff member turning up in the university. My own interdisciplinary field of critical disability studies has emerged as a response to this institutional exclusion of disabled people, offering a distinct understanding of the relationship between disabled people and the university (Meekosha and Shuttleworth 2009, Shildrick, 2012, Goodley 2014, Vaahtera and Lappalainen 2022, Boda 2023, Kulkarni et al 2023). This work reminds us that not only do universities risk perpetuating white privilege they are also in danger of enforcing ability privilege – a modus operandi based upon narrow conceptions of individual ability, isolated achievement, self-sustainability and responsibility – conceptions that promote a logic in which particular kinds of intrinsic human abilities are connected to ‘a *proper life*’ (Vaahtera and Lappalainen 2022, 12; my italics). Universities smuggle in assumptions of ability as self-evident and rarely challenge the neoliberal foundations of ability-as-humanity (Vaahtera and Lappalainen 2022, 9). Ability privilege expects each university student and staff member to be a ready-made, able-bodied-and-minded human being; willing and able to access the normatively constructed physical environment and learning culture of the university. And these expectations feed ingrained ideals associated with academic excellence and intellectual elitism. While universities have become more open – through civic programmes of widening participation, EDI and community outreach – for students and staff to access universities they are still expected to demonstrate evidence of a priori solitary individual academic achievement. Our universities are in danger of idealising able-bodied-and-mindedness (Goodley 2014); marking ability as valued humaneness that feeds into what Wolbring (2008, 2012) defines as *ableism*. This ideology is one that we rarely trouble within the higher education sector, probably because of its entanglement with notions of academic elitism. Our institutions not only welcome clever, gifted and high achieving folk; they also perpetuate a particular kind of ableist logic. This model of the university as an elitist institution geared up for those who can reach the highest echelons of intellectual prowess and individual achievement jars with the university’s civic pretensions. A consequence of an institution being so wrapped up in this ableist logic is that disabled people who rock up in these spaces are not only unwelcome and unexpected; they are known in terms of *pathology*.

Disability studies scholars and disabled activists have long understood the pernicious ways in which disability is socially and culturally constituted as pathological (e.g., Oliver 1990, 1996). To be disabled by society is to be individualised as a person with an impairment of mind and/or body. As Titchkosky (2020, 205) writes, it is common to witness in everyday university discourses, conversations and cultural practices, an understanding of disability that ‘abstracts people from their environments as well as from other people’ to the extent that ‘it remains difficult to locate any version of what disability might be other than lack of function’. Hence, disability as pathology remains a common story re/told with the university. Within medicine, pathology refers to the science of causes and effects of disease. Within the university, disability tends to be understood as a technical problem within a person that requires a solution.^3^ And faced with this pathological constitution of their very being, each disabled member of staff or student is expected to manage their impairment, problem and pathology in the university (Goode 2007). Locating pathology within a person necessitates a personable response. And as disabled students turn up in the university as a pathological problem, then they are constituted as the custodians of their pathologies. Keeping the problem with the disabled individual inevitably invites a conservative response; an untroubling of the ableist architectures and philosophies of university. Disability is often understood as being a problem of the individual. This conception of disability fits well into the medical model of disability or, perhaps more accurately, the medicalisation of disability. Medicalisation is a key component of the many *pathological stories* that locate the problem of disability within the person. In contrast, disability studies scholars and disability activists aim to lift disability outside of the body or mind and place it within the many human and non-human relationships, cultural modes of production and social networks that make us human beings. Rather than understanding disability as a problem to be pathologised – diagnosed, treated and cured through rehabilitation or normalisation (Toro, Kiverstein and Rietveld 2020) – we are encouraged by our disability studies comrades to consider disability as an opportunity to rethink how we might all exist in the world in more equitable ways. Boda (2023, 114) suggests that when disabled staff and students enter the university, they engage in a form critical praxis living, naming and describing their lived realities ‘beyond assumed incompetence’.

Thus far in this paper, I have sought to sketch out the colonial and pathological character of the university which has incubated forms of white and ability privilege. I have also recognised two examples of student activism that, each in their own way, seek to confront privilege within the university. While colonialism/white privilege and ableism/ability privilege clearly overlap, they each have their own distinct historical, cultural, social and material origins. They should not be conflated. Earlier in this paper, I recognised Tuck and Yang’s refusal of decolonisation as a metaphor. And yet, as I start to think about the kinds of words we might use for ridding universities of ability-privilege and ableism, I find myself engaging with metaphor. Titchkosky (2015, 1) argues for the creative potential of disability metaphor to open up ‘the imagination to the possibility of new worlds since it is more than a diagnostic signifier of already dead ones’.^4^ I would like to propose in this paper that we work with the novel metaphor and practice of *depathologisation* in order to adhere ‘to the idea that the meaning of disability is to be found in *something other* than assimilation’ (Titchkosky 2020, 208; my italics). Just as members of the Queer Disability Studies network have worked with the idea of ‘(de)pathologisation’ in their consideration of theory and practice at the border of trans and disability studies,^55^, I seek to develop depathologisation in conversation with decolonisation.

The contemporary push for EDI opens up spaces of critical debate and reflection about the wider aims, responsibilities and accountability of the university. In contrast, white and ability privilege close down debate and maintain the exclusionary character of the university. One wonders if the university can ever be *for* disabled and BAME people when the institution consistently perpetuates systemic white and ability privilege. EDI risks becoming a policy distraction if we fail to challenge the ableist and colonial architectures of the university. In sitting with – and hopefully challenging – white/ability privilege within universities, we connect those who have brought together postcolonial and critical disability studies scholarship. Recent examples of this scholarship have discussed linkages between colonialism and ableism (Grech 2015); disability and development (Chataika 2012); racialisation and disability categorisation (Soldatic 2015); discourses of race and disability in historical accounts of institutionalisation (Altermark 2017); the collusion of whiteness and ability in constitutions of coloniality (Baker 1999) and the generative bringing together of decolonial and disability justice perspectives (Masitera 2020). This paper builds on this intersectional work to think with and across two modes of engagement, decoloniality and depathologisation. Before developing this analysis let us focus our discussion with reference to a new programme of research.

## A research programme

*Disability Matters* is a major new six year pan-national programme of disability, health and science research, funded by a Wellcome Trust Discretionary Award that began in September 2023. While the work of *Disability Matters* focuses on health research, it is broadly interested in the shape, culture, conventions and character of universities; which are key sites for research and innovation. *Disability Matters* brings together disabled academics, researchers and disabled people’s organisations from five countries: Australia, Canada, India, Singapore and the United Kingdom. While the principal investigator (and author of this paper) is a non-disabled person, all the other co-investigators are disabled academics. *Disability Matters* has a grand ambition: to transform health research and research environments through a paradigm shift to disability as the driving subject of inquiry. We want to develop anti-ableist and anti-disablist approaches that promote inclusive research cultures particularly in universities, broaden health research priorities, innovate research methodologies, generate positive disability representations and cultivate a new generation of equitable health researchers across five countries. The country sites represented in the programme capture diverse national/cultural perspectives of disabled people across high/middle income nations across four continents. In thinking of depathologisation and decolonisation for this paper, I have in mind the potential productive impacts of *Disability Matters* on my own context, a British university.

*Disability Matters* is a project conceptualised in the contemporary moment; a time where universities are finally engaging, so it seems, with questions of EDI. Our programme concerns itself with disability and in particular the ways in which health research (and research across many disciplines of STEAM [Science, Technology, the Arts and Maths subjects], medicine and the social sciences) tend to adopt disability as a passive object of intellectual curiosity, empirically investigate disability as a chronic illness or understand disability in terms of impairment or pathology. Too often disability exists as an ‘absent presence’ – a problem that is present (as a problem to be solved) but also absent (as a research colleague or scholarly authority) where disabled health researchers are conspicuously absent (Blume, Galis, and Pineda 2014, Thomas 2007, Titchkosky 2011, Thomas 2021). University research often ignores the specificities of disabled people’s lives and the inequalities that they endure as a consequence of disabling systemic factors. Poor, working class, female, LBGTQ+ and black disabled people are particularly at risk of being forgotten. *Disability Matters* aspires to address these omissions and generate transformative EDI knowledge to support the university, science, research and health sectors to challenge ableism and disablism in their practices and cultures. *Disability Matters* is built upon a number of distinct pan-national research projects, running in parallel, each addressing a distinct research question. Two of these questions include,

- How does the presence of disability enable more inclusive health research environments?
- What transformative knowledge pertaining to equity, diversity and inclusion can be generated through a focus on anti-ableist and anti-disablist practice?

A distinct phase of our programme – *environments* – deploys a host of methodologies to excavate the ways in which universities are inclusive and exclusionary environments for disabled researchers. First, we will undertake a critical policy review of guidance, policy and strategy attending in particular to policies pertaining to equality, diversity and inclusion and researcher development across governments, HEIs, funders and research organisations. This review will also help us to identify recommendations, strategies and capture consensus and conflict relating to equality work. We will ask; how are social actors within university environments constituted through various discourses? To what extent do university policies and practices expect disabled staff and students to turn up to the campus? The onus is on the very practical ramifications of disability discourses; that hold back or release the potential disabled people.

Second, to investigate the practice of health research cultures and university environments – and their potential to include disabled people – we will design and deliver an *Online Survey* of 500 research leaders (from Pro/Vice Chancellors to Directors of Research). We want to understand how these university leaders understand equality, diversity and inclusion, how they conceptualise their role in facilitating and taking forward EDI agendas and to explore with them how they understand their university’s historical relationship with disability. Third, to access first-hand accounts we will carry out *Narrative Interviews* with 200 disabled researchers and academics (40 informants per country spanning early, mid and late career positions). We will spend time crafting a set of questions which will invite considered and critical reflection on the part of disabled researchers. Fourth, disabled colleagues of *Disability Matters* will engage in auto-ethnography. If ethnography is the anthropological method for interrogating the workings, conventions and rules of culture, then auto-ethnography is a very personalised methodology that considers one-self and our own very personal experiences of the particular culture that we inhabit.^6^ Hence, we will access the retrospective and ongoing critical commentaries of our disabled researcher team members as they critically reflect on their own experiences of the university. Across the different methodological phases, we will deploy a host of thematic and discourse analyses, depending on the kinds of sub-questions we might want to ask.

At the time of writing, the *Disability Matters* team are in the provisional phase of planning these empirical work packages. Even at this early stage of the programme, I find myself thinking about my own university and whether or not it is ready to truly embrace disabled staff and students. The two examples of student activism presented earlier provide stark reminders of the unwelcoming nature of universities but entry points into theorising the study of inclusive university culture. As Hammond (2018) has discussed, even before implementing fieldwork, researchers are already working through theoretical perspectives that might hold value, significance and purchase. In this spirit, this paper sketches out some provisional theoretical approaches of decolonisation (drawing on postcolonial scholarship) and depathologisation (developed through the application of disability studies scholarship) in relation to the university. In sitting with decolonisation and depathologisation there is no suggestion that they are the same. Instead, one might view them as distinct and potentially complementary practices that challenge universities to consider what they are far; in ways that challenge institutional racism and ableism. I offer some provisional and anticipatory thoughts in relation to the potentially generative links made by bringing decolonising and depathologisation into conversation.

## Sitting with decolonisation to depathologise the university

As a disability studies researcher based in a British university, I am keenly aware of the criticisms directed at WENA disability studies. These flaws include, following Meekosha (2008, 2011), claims to universality (what happens in the Global North should happen in the Global South), a reading from the Metropole (a methodological projection of ideas from the centre into the periphery) and an emphasis on the importance of Northern feudal/capitalist modes of production (with an accompanying ignorance and grand erasure of indigenous modes of living of the South). The inclusion of disabled research partners across the UK, Australia, Canada, India and Singapore has the potential to move *Disability Matters* from a EuroAmerican anchoring to a more complex and nuanced pannational location. Decolonising *Disability Matters* involves not repeating the mistakes outlined by Meekosha and attending proactively to the different ways in which disability is constituted across various landscapes. To paraphrase Bhambra et al (2020), our programme team aspires to recover and create alternative research aspirations free from colonial legacies and institutional racism. We hope to build a research programme for the public good of critical thinking, educated deliberation and informed citizenship. Having said all that, it is important to acknowledge the different kinds of colonial histories that each of the partner organisations will bring with them.

One decolonising desire of *Disability Matters* is to adopt a dynamic, inclusive and diverse approach to the recruitment of disabled participants. Recruitment can be used here in its orthodox research sense – to invite people to participate in various research activities of the programme of work – but can also be used more expansively to invite disability into research. We will actively seek to recruit a wide representation of disabled adult participants including those with physical and sensory impairments, long-term mental health issues, developmental disabilities and neurodiversity. We will work with disabled researchers and disabled people’s organisation collaborators who have a proven track record of working with diverse groups. Cultural and ethnic diversity will be captured by the national locations of our research. Singaporean research projects will permit access to participants of Chinese, Malay, Indian and Eurasian background while in India we will seek representation from Indo-Aryan, Dravidian and Tibeti-Burman communities. Australian and Canadian research sites will permit access to indigenous and first nations disabled people and the UK site will build on strong relationships with people with labels of learning disabilities and neurodiversity. We have financially budgeted for disability access to deliver on these inclusive aspirations (e.g., sign language interpreters, captioning, easy read). We recognise that ‘youth’ and ‘young adulthood’ age ranges have been expanded in some countries and are therefore interested in bringing in younger disabled adults (18+). While previous research has suggested that age is a confounding variable when combined with disability we will ensure that disabled elders also participate in our research. Finally, our intersectional understanding of disability means investing in disability communities that experience the most extreme forms of marginalisation. We are keen to ensure the involvement of disabled participants from LGBTQ+, black, first nations, neurodiverse, poor and working class communities and those in rural as well as urban localities.

These inclusive aspirations offer a further invitation; to engage with decolonisation and specifically the work of Zondi (2022). The first element relates to embracing our relational selves. Zondi (2022, 237) demands we ‘embrace in earnest and in practice the ways of being long provided for in indigenous paradigms of being, such as ubuntu’. Many diverse communities have always historically engaged with relational ways of being with one another in the world. Collaborating with and alongside disabled people from diverse communities has the potential to bring us into these new ways of relating. Colonialism and racism are perpetuated through practices of division where some human beings are valued and others devalued. Turning to relationality encourages reconnection and reaf-firmation. *Disability Matters*’ commitment to inclusive recruitment can be read as an example of desiring the embrace of relationalities between disabled people across diverse communities.

Zondi’s second element emphasises a mutual recognition of the humanity of others. This entails ‘being and doing human as a process of restoring, enriching and reinforcing the humanity of others, through our speech, the ways we relate to others, and the design of human systems’ (Zondi 2022, 238). ‘Speech’ and ‘speaking’ as practices could be read as potentially exclusionary; particularly to those disabled people that might not use speech as a key mode of communication. Perhaps writing might be a more inclusive term. So, *how* we write of disability, to *whom* we write with about disability and *where* we write to one another about disability are just some of the considerations to keep in mind in researching disability. Indeed, including marginalised disability communities requires a commitment to recognising the mutual humanity of one another.

The third element – a decolonising commitment to communalism – is understood principally ‘as a way of living, of co-existing and working with others. It requires conscious efforts to function in ways that build communities and communal practices instead of perpetuating esoteric individualism that breaks human bonds’ (Zondi 2022, 239). Research should not be extractive nor individualising but proactive and connecting. The *Disability Matters* team will spend time and resources to ensure that disabled people from different national spaces can commune with one another beyond the aims and timeframe of the research programme. This involves breaking down non/academic boundaries and recognising that knowledge produced in disability communities has as much value if not more than the knowledge that is produced in the university.

The fourth element endeavours to ‘achieve human excellence with humaneness’ (Zondi 2022, 239). Any research encounter should commence and end with a commitment to working humanely with one another across disability communities. Co-production and co-creation methods are forms of inquiry that move us from the usual mode of ‘disability as pathological object’ to ‘disability as driving subject’ where disabled researchers are front and centre of research. Inserting humaneness into the research encounter is not without challenges. Many disabled people are sick and tired of being researched. And the disability community has a deep distrust of research – particularly university research – because of a history of pathologising research that has helped feed deficit models of disabled people. Being humane in research involves checking in throughout the research production process that participants, partners and recipients of research are capacitated and not erased by research.

The fifth element of Zondi’s (2022, 239) framework relates to going beyond knowledge production. The critical interventions of disabled researchers, activists and artists push us to consider how we might think through and with decolonisation to ‘support one another to create new knowledge that is anti-bourgeois, anti-colonial, anti-imperial, anti-global and, may we add, anti-ableist and anti-disablist’ (Zondi 2022, 232). Similarly, *Disability Matters* wants to change the university, to undo and contest pathological conceptions of disability that are rife in all of our disciplinary domains. While depathologisation is a metaphor, it is also praxis (as Tuck and Yang 2012 have advised). One of the many knock-on effects of pathologising disability is the promotion of disablist cultures (where people with impairments are treated like second class citizens in the university) and the maintenance of ableist cultures (which assume the presence of non-disabled people and those who fit the ableist imperative of higher education). Universities are elitist institutions founded upon ableist foundations. Hence, academically gifted individuals are given access to the university. Any attempt to water down this reliance on ability and achievement could be interpreted as undermining the elitist aspirations of the institution. This discussion is bringing us back, yet again, to the question, what is the university for? And following Lalu (2019) this question relates to who the university serves. If universities are civic universities – and most university websites indicate that they are – then the university should welcome *all* members of the communities that it purports to serve. Ensuring that universities are inclusive, equitable and diverse requires an interrogation of the assumptions, philosophies and spaces that constitute the modern-day university (Cox et al 2022). One of these is ability privilege which refers to the benefits reaped by typically abled individuals afforded in relation to their position within the hegemony (Bialka and Morro 2017, 18). Here, as above, I need to pause, to consider my own ability privilege. And while any attempts to transform the university must be led by disabled colleagues, non-disabled folks just like me are required to respond and engage. By drawing in Zondi’s and others’ work on decolonisation – and in contemplating the aspirations of *Disability Matters* – I am finding a clearer sense of what the idea of depathologising the university might mean, what it provokes and what it evokes. Here, I am thinking with *disability as authority*. Too often, disability occupies an ‘absent presence’ in the university (Titchkosky 2011). Disability is present as a problem to be solved. Disability appears as a problem of the student, academic or professional services colleague. Many more people are prepared to disclose their disability status. Various reasonable and workplace adjustments are put in place to help support the student. So disability is present in the academy but too often only as a metaphor for failure and pathology (Titchkosky 2015). Disability is also absent as an authority, as a colleague, as a resource and as an opportunity. In order to address this absent presence, we must centre disability in our curricula, in our classrooms, in our EDI policies. We should ask, how can disabled people drive forward change and transformation in our universities? One response is to consider disability as authority rather than a problem and to ask: are university cultures embracing the presence of disability?

Depathologising the university urges a cultural reconceptualisation of disability and the promotion of disability studies literacy in the university. When university courses promote only pathological narratives of disability then they only tell partial stories about disability and risk creating epistemic injustice (Peña-Guzmán and Reynolds 2019). Epistemes are forms of knowledge on which we draw to make sense of the world. When epistemes about disability are narrowly framed around ideas of pathologising then disabled people risk being understood purely in terms of these knowledge systems. This does not do justice to other disability epistemes – often developed by disability studies scholars and activists – that have politicised the lives of disabled people. Disability studies must be included in all course curricula so that texts, teaching and learning can be depathologised. This means disabled people driving course development, engaging with the rich literature of disability studies and ensuring that disability studies is used in reformulating how different disciplines understand disability. This requires forms of critical pedagogy, using the labour, research and teaching of academics to reinvest the university with knowledge produced by disabled people (Lynch, Simon and Maher 2023). And this work might bring about more epistemic justice through us all building our disability studies literacy together.

A commitment to epistemic justice encourages us to collectively confront systemic ableism and disablism. Following Lee (2022, 2023), structural ableism is not only evident in the ways in which academics are expected to access the university (walking up the steps to access the higher echelons of the ivory tower) but also in the emphasis on the self-contained, funded, published, performative academic ideal type so valued by the university. And this idealisation is always racialised, classed, sexed or gendered. Disabled and black colleagues face complex intersectional issues in terms of academic promotion and also in terms of surviving day to day in unwelcoming university contexts. We need to keep in mind the differences between ableism and disablism rather than to conflate these terms. Disablism is something distinctly experienced by disabled people. When a wheelchair user cannot access the tiered lecture hall then they experience disablism. Ableism is a practice that impacts on all of us. And depathologisation should be engaged with addressing both of these practices in tandem.

Engagement, in this sense, might well involve revolt; ‘an opposition to already established norms, values and powers which ‘serves as a method to question what an activist university is and might be’ (Arndt 2021, 529). Arndt (2021, 528) depicts the university as a vibrant living, throbbing assemblage of beings, policies and practices that are closely and often indiscernibly entangled.

Revolt provokes a rethinking of both the aim and method of constructing, enacting and debating policy in the university assemblage … tiny revolts calls for inner diffractive thought processes, where individual academics might respond to policy change, for example, by questioning and re-questioning, reading and re-reading to challenge and shift, rather than take for granted, their orientations (Arndt 2021, 530)

Moving from large-scale to tiny revolt should not be confused with a move from radicalism to reformation. For university actors to adopt disability as authority requires a reorientation from disability as pathology to disability as provocateur. Understanding disability as an opportunity to rethink the university – rather than a problem that has to be managed and accommodated in the university – constitutes a leap of faith for many people because pathological ideas of disability are so centrally located in our culture and society. Disability’s potential to promote revolt in the university has never been more timely than in these (so-called) post-pandemic times. We should never forget Covid-19’s devastating impact on the lives of disabled people. Many disabled people died as a consequence of this virus and many others too were not treated as human beings worth saving by their governments and health systems. And yet, alongside this death-making, COVID-19 radically disrupted the workings of universities in some potentially very positive ways, not least with the rapid move from the offline to the online, especially during national lockdowns. An awful irony occurred for disabled people. While many disabled folks had argued for years for more flexible kinds of working – such as those that embrace online platforms – it took a global pandemic to move university labour from fixed notions of face-to-face office-based labour to online home working. For some disabled people they at last could work in ways that suited their ways of being in the world. While I am not uncritically putting forward the idea that the online is a panacea to problems of access, disability has always provided opportunities for rethinking the operations of the university. It is telling, though, how universities have often ignored disability as authority. Some disabled academics have fought with their universities to remain at home teaching online, convening conferences online, writing and researching online. Other colleagues value being back in the university – for some days – but welcome flexibility to work from home. As Arndt (2021, 537) puts it, ‘by re-thinking the disruptions that the virus has wrought on ‘established norms, values and powers’, tiny revolts perhaps are the future for making meaning of this ‘new normal’ in the activist university’.

A final theme pertaining to depathologisation is the need for intersectional engagement. We must hold on to Crenshaw’s (1991) original conceptualisation of intersectionality that emphasised the tensions as well as complementarities that occur when different kinds of politics meet. There will be times when decolonisation and depathologisation butt up against one another. We have to take these moments of tension seriously and to consider how we might work with them together within the university. This will involve constant dialogue between Black and disability studies as they challenge one another (Goodley et al 2021). We need to liaise with comrades who have asserted that much of what passes as disability studies implicitly assumes whiteness and risks white-washing the phenomenon of disabled people (Dunhamn et al. 2015). Simultaneously, we need to ensure that any call for anti-racist transformation in the university does not call upon disabling metaphors in order to make its case (Titchkosky 2015). Finding a shared politics of intersectional engagement is never easy, but there are plenty of colleagues across many universities whose work we can draw upon to inform our work.

## Conclusion

This paper has explored the ways in which the very notion of the civic university – an institution that serves the needs of its local, national and international communities – is compromised by colonial heritage and ableist architecture that promulgate contemporary forms of white and ability privilege. While universities might boast about their EDI policies and practices, these interventions risk falling into forms of equity tourism when they fail to redress ability and white privilege. Our discussion then moved on to consider a new six year programme of research – *Disability Matters* – and used this new study as a research site for considering two different though potentially generative modes of engagement, decolonisation and depathologisation. While neither should be conflated with the other, we identified a number of overlapping intentions and interventions that might help to contest racism and ableism. Unless universities consider their broader responsibilities and dominant cultural practices, they risk being elitist, ableist institutions that will only ever superficially engage with EDI. As we enter a period of post-pandemic life, it is incumbent upon us all to rethink how we constitute the higher education institutions in which we work. For Arndt (2021) COVID-19 can be viewed as an opportunity to reorient towards the university. One reorientation could involve regenerating the university’s relationship to its wider communities. Disability has much to offer here precisely because disability is inherently disruptive and productive. One personal highlight for me, during the COVID-19 lockdown, was the popular release and response to the Netflix documentary *Crip Camp*, a timely reminder of the interconnections of Black and Disability politics in North America (Netflix 2020). This beautiful film captured the radical work of disabled activists such as Judith Heumann and James Lebrecht as they came together during a summer camp to hone their politics and create their own disability commons. Their stories captured the ways in which disability and black civil rights activists learned from one another to contest wider disablist and racist society. Over time their campaigns transferred from the summer camp to the university campus. *Crip Camp*’s transition from the summer camp to the university is a move we might keep in mind when contemplating the transformation of higher education universities. Much is made of the civic university, not least, by universities themselves. But any civic engagement has to be enacted with a commitment to anti-racism, anti-ableism and anti-disablism. And any attempt to depathologise the university must be undertaken in concert with its civic partners – disabled people’s organisations – in and outside of the university.

## Notes

1 In grounding this paper in a discussion of the British university I am acutely aware of the problematic nature of keeping my discussion framed within the global north context. Indeed, my analysis is in danger of only illuminating those material and epistemic realities of the global north, engaging with researchers and scholars in this context and ignoring powerful discussions amendating from the Global South. I accept all of these criticisms and am thankful to one of the anonymous reviewers for urging me to sit with this inherent limitation of my writing. In response, the paper seeks to bring in scholarship from the Global South not least in relation to discussions of decolonisation.

2 BAME is a contentious term deployed in the UK to recognise Black and Minority Ethnic people. This paper uses this term in recognition of its wide deployment in the British context but also recognises other terms such as Black, Person of Colour and BIPOC (Black, Indigenous and People of Colour) to recognise the preferred terms of scholars whose work is drawn upon.

3 Thanks to Rod Michalko (pers. comm.) for this phrase.

4 What is interesting about metaphor is how readily it is used in activist and critical literature – note the ways in which, for example, the student campaigner users disability metaphor to further a decolonial project, referring as they do to an ‘Imperial blind spot’ in the Khomami (2015) article presented above

5
Queer Disability Studies Network (2021)

6 For a wonderful example, check out the work of Kasnitz (2020)
